# The Risk of False-Positive Serological Results for Paratuberculosis in *Mycobacterium bovis*-Infected Cattle

**DOI:** 10.3390/pathogens10081054

**Published:** 2021-08-19

**Authors:** Anna Didkowska, Monika Krajewska-Wędzina, Daniel Klich, Kinga Prolejko, Blanka Orłowska, Krzysztof Anusz

**Affiliations:** 1 Department of Food Hygiene and Public Health Protection, Institute of Veterinary Medicine, Warsaw University of Life Sciences (SGGW), 02-776 Warsaw, Poland; kinga.prolejko@vp.pl (K.P.); blanka_orlowska@sggw.edu.pl (B.O.); krzysztof_anusz@sggw.edu.pl (K.A.); 2 Department of Microbiology, National Veterinary Research Institute, 24-100 Puławy, Poland; kappa2@wp.pl; 3 Department of Animal Genetics and Conservation, Institute of Animal Sciences, Warsaw University of Life Sciences (SGGW), 02-786 Warsaw, Poland; daniel_klich@sggw.edu.pl

**Keywords:** cross-reaction, cattle, diagnosis, ELISA paratuberculosis, tuberculosis, MTBC, *Mycobacterium avium* spp. *paratuberculosis*

## Abstract

Both bovine tuberculosis (BTB) and paratuberculosis (paraTB) continue to cause significant economic losses in cattle breeding; in addition, their etiological agents have zoonotic potential. Although the diagnostics of both diseases are still being improved, problems still remain, such as the potential for cross-reactivity to the antigens used in tests. The aim of the present study was to confirm whether animals known to harbor *Mycobacterium bovis* antibodies are at increased risk of yielding positive results in paraTB serotesting and, additionally, to verify the accuracy of three commonly used methods for confirming *M. bovis* infection: ELISA, the tuberculin skin test (TST), and the presence of gross lesions. Material was collected from 98 dairy cattle suspected of BTB due to TST-positive results. During postmortem examination, gross lesions were assessed visually. Blood, lymph nodes, and TB-suspected organs were collected. Serum was obtained from the collected blood and tested serologically for TB and paraTB. The tissues underwent standard microbiological testing for *M. tuberculosis* complex. Among the 98 TST-positive individuals, tuberculous gross lesions were detected in 57 (58.1%), MTBC were isolated in 83 (84.7%), and the ELISA test was positive for 21 (21.4%). None of the lesions characteristic for paraTB were detected. The chance of obtaining a positive TB result by ELISA was seven times higher using the ELISA-paraTB method; hence, there is a significant risk of obtaining false-positive serological results for paraTB in *M. bovis*-infected cattle. However, the hypothesis that infection of *M. bovis* or prior TST performance may have boosted the host immune response and therefore increased the sensitivity of the paraTB-ELISA cannot be excluded.

## 1. Introduction

Mycobacterial infections, both bovine tuberculosis (BTB) and mycobacteriosis, continue to threaten the health of both animals and humans [1,2]. One of the most widespread nontuberculous mycobacterial (NTM) infections is paratuberculosis, also known as Johne’s disease. Its etiological agent, *Mycobacterium avium* spp. *paratuberculosis* (MAP), causes granulomatous enteritis. Paratuberculosis (paraTB) mainly occurs in ruminants and may cause significant economic losses for breeders [3].

As the diagnosis of mycobacterial infections, both those caused by *Mycobacterium tuberculosis* complex (MTBC) and NTM, still presents difficulties, there is a need to improve diagnostic tests [4,5]. One such difficulty is presented by the influence of cross-reactive immune responses towards different mycobacteria [6]. Many studies have found MAP infection to impact BTB test results [7], while others report no significant effect [8]. It is also known that exposure to environmental NTM can reduce the specificity of *M. bovis* diagnostic tests [9]. However, only a few studies [10,11] have examined the inverse relationship: the effect of MTBC infection on MAP diagnostic results.

The aim of this study was to determine the effect of *M. bovis* infection on the results of serological testing for paraTB in cows, and to compare the accuracy of the tuberculin skin test (TST), the occurrence of gross lesions, and ELISA testing for diagnosing MTBC in cattle.

## 2. Results

In TST, all animals had a positive reaction for bovine tuberculin and a negative reaction for avian tuberculin. Among 98 TST-positive individuals, tuberculous gross lesions were detected in 57 (58.1%), MTBC were isolated in 83 (84.7%), and the TB-ELISA test was positive for 21 (21.4%). The gross lesions were mostly localized in one or two lymph nodes of the thoracic region; tuberculous lesions were confirmed in one individual, in both the liver and peritoneum, and for two others, in the lungs and pleura. No lesions characteristic of paraTB were detected in the intestines or mesenteric lymph nodes.

None of the other methods (TST, gross lesion occurrence, or TB-ELISA) yielded similar results to the reference method, that is, MTBC isolation (Table 1). The sensitivity and specificity for the TST were respectively 100% and 0% (with reference to MTBC isolation). Gross lesion occurrence demonstrated 67.5% sensitivity and 100% specificity, and TB-ELISA demonstrated 24.1% sensitivity and 93.3% specificity (with reference to MTBC isolation). ParaTB-ELISA demonstrated 37.3% apparent sensitivity and 80% apparent specificity (with reference to MTBC isolation).

The cumulative evaluation of the measures indicates high accuracy for the tuberculin test (84.7%) and the gross lesions method (72.4%). TB-ELISA only demonstrated 34.7% accuracy. Interestingly, the paraTB-ELISA method, not dedicated to TB detection, demonstrated 43.9% apparent accuracy. Neither of the ELISA methods (Wald χ^2^ = 1.613, *p* = 0.204 for paraTB and Wald χ^2^ = 1.956, *p* = 0.162 for TB) nor the gross lesions method (Wald χ^2^ = 0.000, *p* = 0.997) could be used to predict positive results of tuberculosis testing based on MTBC isolation.

**Figure 1:**
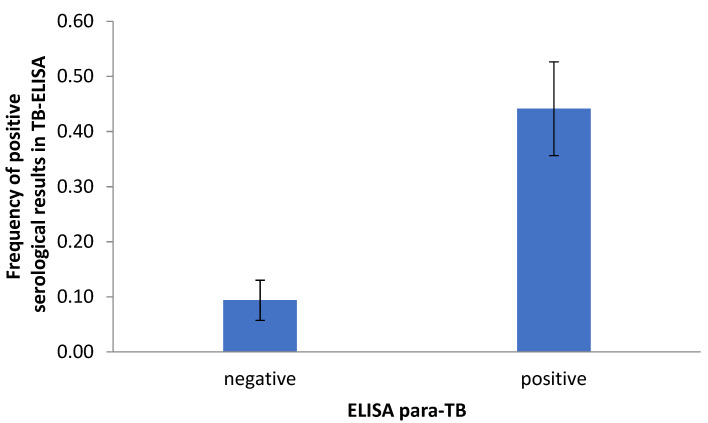
Frequency of false-positive serological results in paraTB-ELISA compared to TB-ELISA.

The TB-ELISA and paraTB-ELISA methods yielded very similar results: paraTB-ELISA demonstrated 71.4% apparent sensitivity and 75.3% apparent specificity compared to the TB-ELISA method, with overall 74.4% apparent accuracy. Positive results of TB-ELISA could be predicted by those of paraTB-ELISA. The chance of obtaining a positive result by the TB-ELISA method was seven times higher when tuberculosis was detected using the paraTB-ELISA (Table 2). Additionally, the frequency of positive results by TB-ELISA was almost five times higher in cases of positive paraTB-ELISA than negative paraTB-ELISA (Figure 1).

## 3. Discussion

Our findings confirm that both BTB and paraTB are difficult to diagnose, and therefore the process requires extreme vigilance and a multifaceted approach; this is in line with previous reports [11,12,13,14,15,16,17]. In addition, our results indicate a significant risk that false-positive serological results for paratuberculosis could be obtained in *M. bovis-*infected cattle. Most studies so far have focused on the problem of detecting false-positive results for MTBC caused by the presence of NTM, particularly environmental mycobacteria [6,17,18]. The cross-reaction is caused by the immune response of the host being primed by mycobacterial agents that are common to MTBC and NTM. For this reason, the reverse case is also possible: false-positive NTM results can be obtained in *M. bovis*-infected animals. This issue merits serious consideration in bovine paratuberculosis and tuberculosis control programs, and additional direct testing should be implemented for seropositive herds.

New methods are being designed to minimize the risk of false-positive results, mainly those based on the usage of appropriate, highly-specific antigens [19,20,21]. It is also believed that some specific antigens may allow differentiation between paratuberculosis and BTB [22]. However, as our findings indicate, some aspects remain unexplained. As the precise antigens used in the paraTB-ELISA test remain a proprietary secret, it is difficult to assess exactly what cross-reactions can occur with MTBC. However, as our study shows, such reactions may occur frequently. Fortunately, increasing numbers of new antigens are being discovered that could potentially be used for the serological diagnosis of paratuberculosis [23].

It should be highlighted that in addition to *M. bovis-*infection, performing a TST can also influence MAP-positive results in tested animals. It is possible that TST could boost the antibody response and increase the sensitivity of paratuberculosis ELISA results [10,24]; this would indicate that at least some of the results were not false positives. However, the limited number of serological studies in Poland have found the seroprevalence of paratuberculosis in cattle to be much lower (1–3%) [25]. As such, it is likely that the paratuberculosis ELISA assay used in this study returned false-positive results, and that cross-reactivity was possible. It should also be highlighted that performing TST can influence not only the paraTB-ELISA results, but also increase the likelihood of positive TB-ELISA. Performing TST before the TB-ELISA test has been found to increase sensitivity because of the amnestic effect [26]. Conversely, apparent sensitivity was found to be very low in studies where serological tests were performed prior to TST, which confirms its stimulating effect [27]. TST can boost response to the MPB83 and MPB70 antigens which are used in the IDEXX test. Interestingly, this correlation was not found for other antigens [28]. However, repeating TST can lead to desensitization and false-negative results [29]. This study has some limitations. Firstly, the animals were not microbiologically tested for MAP (specific media and material were not used); despite this, the GenoType *Mycobacterium* CM Test (Hain Lifescience, Nehren, Germany) also detects mycobacteria from the NTM group, and only MTBC was confirmed. At the moment, there are no scientifically published data on the seroprevalence in cattle in Poland, while our own research shows that that about 11–14% of cattle herds have positive results, which is a much lower percentage than that shown in the present study. Given the relatively low seroprevalence of paratuberculosis in Polish cattle in other regions [25], lack of characteristic clinical signs or lesions for paraTB, as well as lack of positive results for avian tuberculin in TST, it should be expected that this limitation did not significantly affect the results. Although, to the best of our knowledge, there are no precise data on the sensitivity of autopsy as a diagnostic test for paraTB, taking into account the high percentage of positive paraTB serological results compared to the Polish cattle population, the lack of response for avian tuberculin and NTM-negative results in the GenoType *Mycobacterium* CM Test, the lack of gross lesions is another factor suggesting that most of the tested animals had a false-positive reaction in the paraTB-ELISA.

Future experiments should also include feces collection and tests for paratuberculosis based on culture and PCR [30,31,32]. In recent field studies of dairy cows, fecal examination by real-time PCR testing of MAP-specific DNA was found to have 74% sensitivity of culture [33]. In recent studies, peptide magnetic separation PCR test (PMS-PCR) was found to obtain greater sensitivity and lower specificity than ELISA for paratuberculosis diagnosis [34], which indicates that this method has potential.

It should also be noted that our study focused mainly on the effect of MTBC infection on the results of the paratuberculosis serology test. However, other factors such as age, sex, animal type (dairy vs. beef), or breed may also affect the results [35,36], which was not considered in the present study. The manufacturer indicates that the specificity of the PARACHEK^®^ 2 *Mycobacterium paratuberculosis* Test Kit for Cattle (Thermo Fisher Scientific Inc., Waltham, MA, USA) is 99%, with a sensitivity of 60–80%. However, we suspect that these figures were acquired through testing on random animals and not (as in our experiment) on those suspected of carrying BTB.

In addition, in the present study, the tests for the diagnosis of BTB were evaluated against the results for microbial culture (i.e., the most commonly used method for confirming BTB from TST-positive slaughtered cattle) [37], and no direct PCR was conducted on the samples. The results of the occurrence of gross lesions test were generally not surprising (specificity 100%), but the sensitivity (67.5%) was slightly lower than in previous studies [38]. The ELISA test for tuberculosis demonstrated low sensitivity (24.1%) despite prior sensitization with the tuberculin test. Perhaps the time between sampling (i.e., 30 days on average) was too long [39,40]. However, previous field studies have shown very similar sensitivity and specificity of the ELISA IDEXX test—26.74% [41].

## 4. Conclusions

None of the tested methods demonstrated high accuracy towards *M. bovis*, and a significant risk of obtaining false-positive serological results for paratuberculosis was observed in *M. bovis-*infected cattle. However, the hypothesis that infection by *M. bovis* or the performance of TST may boost the host immune response, and therefore increase para-ELISA sensitivity, cannot be excluded. In fact, the TB-ELISA and paraTB-ELISA tests showed consistent results, indicating the need for caution when interpreting positive and negative test results. Further studies are needed in this field.

## 5. Materials and Methods

### 5.1. Material

The material was collected from 98 dairy cattle (*Bos taurus*) suspected of BTB due to positive comparative cervical tuberculin (CCT) test results. The animals originated from four regions of Poland: northwest, east, central, and north. All animals had been slaughtered due to positive TST results, in accordance with regulations of the Polish Chief Veterinary Officer [42]. On average, the blood was collected 30 days after performing TST.

In the slaughterhouse, blood was collected from the jugular vein of each animal into 9 mL tubes with clotting activator and centrifuged within 24 h. The obtained sera were stored at −70 °C.

Postmortem examination was conducted in accordance with the rules of sanitary slaughter, outside of the main slaughter line. A detailed postmortem examination was carried out, including the assessment of the digestive system in terms of lesions typical for paratuberculosis. During the postmortem examination, gross lesions were assessed, and the following material was collected for microbiological examination: the hepatic hilus as well as the retropharyngeal, mandibular, tracheobronchial, mediastinal, and the supramammary and mesenteric lymph nodes. Additionally, organs with any kind of gross lesions were collected for microbiological examination. 

### 5.2. Serology

Before starting the laboratory procedures, the serum samples were defrosted and brought to room temperature, which has been confirmed to not have any influence on the results of the ELISA test [43]. They were then subjected to two ELISA tests: PARACHEK^®^ 2 *Mycobacterium paratuberculosis* Test Kit for Cattle (Prionics AG, Schlieren-Zurich, Switzerland) (paraTB-ELISA) and IDEXX *M. bovis* Ab ELISA (IDEXX Laboratories, Inc., Westbrook, ME, USA) (TB-ELISA). The first test detects specific antibodies against *M. paratuberculosis,* and the second detects the presence of IgG antibodies against *M. bovis* antigens (MPB83 and MPB70). Both tests were conducted in accordance with the manufacturers’ instructions.

Briefly, for the *Mycobacterium paratuberculosis* Test Kit for Cattle PARACHEK^®^ 2, the serum samples were first diluted and incubated 1:20 in buffer containing *Mycobacterium phlei* to remove cross-reaction and transferred to a test plate (incubation: 30 min, room temperature). After washing with wash buffer, conjugate was added to each well and incubated for 30 min at room temperature. After washing, the enzyme substrate was added. After the plate incubation (15 min, room temperature), the enzyme stopping solution was added. The absorbance for each well was read at a wavelength of 450 nm with an EPOCH spectrophotometer (BioTek Instruments, Inc., Winooski, VT, USA). The criteria given by the manufacturer were fulfilled: the mean corrected value of positive control greater than 0.500: ODPC > 0.500, and the mean-corrected value of positive control to negative control ratio greater than 5: ODPC/ODNC > 5. The results were considered as positive if the sample %P was greater than 15% (%P = (ODsample − ODNC/ODPC − ODNC) × 100%).

The IDEXX *M. bovis* Ab ELISA was performed as follows. After dilution (1:50), the samples were dispensed into wells on a plate. The plate was incubated for 60 min (room temperature) and then washed. After adding conjugate, the plate was incubated again (30 min, room temperature) and subsequently washed. Following this, the TMB substrate was added, and incubation was repeated. Stopping solution was then added and the optical density (OD450) was measured with an EPOCH plate reader. The result was expressed as the value of sample (S) divided by the value of the positive control (P). The interpretation criteria were adopted in accordance with the manufacturer’s instructions: a negative result was associated with S/P ratio less than 0.30, and a positive one with S/P equal to or greater than 0.30.

### 5.3. Mycobacterial Isolation

Mycobacterial isolation was conducted as described previously [44]. Briefly, the tissues (i.e., lymph nodes and other organs) were removed from the animal and decontaminated with 5% oxalic acid. After homogenization, the sediment was plated on Stonebrink and Petragnani media in triplicate and incubated at 37 °C for 12 weeks, and was checked every seven days. If colonies appeared on the media, the DNA was isolated with the GenoLyse Isolation Kit (Hain Lifescience, Nehren, Germany) and classified to MTBC by using the GenoType *Mycobacterium* CM Test (Hain Lifescience, Nehren, Germany).

### 5.4. Statistical Analysis

As microbial culture (MTBC) is considered the most sensitive method of those used in this study [45], it was considered as the reference in the statistical analysis. Therefore, the sensitivity, specificity, and accuracy (defined as (true positive + true negative)/(total positive + total negative)) of the tuberculin skin test, TB-ELISA, paraTB-ELISA, and gross lesion methods were evaluated with reference to the MTBC method. Note that as indicators for the paraTB-ELISA do not truly represent the sensitivity, specificity, or accuracy of MAP, they are apparent. Similarly, TB-ELISA was treated as a reference method for the paraTB-ELISA, and the indicators are also apparent. It must be considered that in the present study, sensitivity, specificity, and accuracy are only intended to compare methods, and cannot be used for the individual methods in isolation.

The results of the ELISA (TB and paraTB) and gross lesions occurrence methods were subjected to logistic regression analysis to determine whether they were associated with the MTBC method. In each model, the dependent variable was the MTBC result, with a positive result indicated as 1 and a negative result as 0. The results of the other methods were included as separate independent variables in each regression, with the same numerical designations as in the MTBC method. Similarly, the TB-ELISA and paraTB-ELISA methods were compared by logistic regression, where the TB results were used as the dependent variable and the paraTB results as the independent variable.

## Figures and Tables

**Table 1:** Tuberculosis detection by various methods in relation to MTBC isolation (−: negative, +: positive).

Reference Method	Compared Methods							
	Tuberculin Skin Test		TB-ELISA		ParaTB-ELISA		Gross Lesions	
MTBC isolation	−	+	−	+	−	+	−	+
−	0	15	14	1	12	3	15	0
+	0	83	63	20	52	31	27	56

**Table 2:** Tuberculosis detection by TB-ELISA and paraTB-ELISA (−: negative, +: positive), and statistical evaluation of the potential for paraTB-ELISA to predict detection by TB-ELISA by logistic regression (Wald χ^2^ and *p*-values for independent variable and intercept).

Reference Method	ParaTB-ELISA		Logistic Regression Results
TB-ELISA	−	+	B_var_ = 2.03, (SE = 0.55), Wald χ^2^ = 13.622, *p* < 0.001, OR = 7.63 B_0_ = −2.27, (SE = 0.43), Wald χ^2^ = 27.986, *p* < 0.001, OR = 0.1
−	58	19	
+	6	15	

## Data Availability

Data can be found in the Department of Food Hygiene and Public Health Protection, Institute of Veterinary Medicine, Warsaw University of Life Sciences (SGGW), Warsaw, Poland; and the Department of Microbiology, National Veterinary Research Institute, Puławy, Poland.
